# Tuberculosis Infection Screening Recommendations for Targeted Immunotherapies: Comparison of US Prescribing Information, Clinical Resources, and Quality Measures

**DOI:** 10.1093/cid/ciaf628

**Published:** 2025-11-19

**Authors:** Matthew T Murrill, Gustavo E Velásquez, John D Szumowski, Allison Phillips, Annie Kim, Jinoos Yazdany, Eric T Roberts, Anand R Habib, Haiyan Ramirez Batlle, Jorge Salazar, Daniel J Minter, Janice K Louie, Joel D Ernst

**Affiliations:** Division of Hospital Medicine, University of California, San Francisco, San Francisco, California, USA; UCSF Center for Tuberculosis, Institute for Global Health Sciences, University of California, San Francisco, San Francisco, California, USA; UCSF Center for Tuberculosis, Institute for Global Health Sciences, University of California, San Francisco, San Francisco, California, USA; Division of HIV, Infectious Diseases, and Global Medicine, University of California, San Francisco, San Francisco, California, USA; San Francisco Department of Public Health Prevention and Control Program, San Francisco, California, USA; San Francisco Department of Public Health Prevention and Control Program, San Francisco, California, USA; Department of Pharmacy, University of Washington Medicine, Seattle, Washington, USA; Division of Rheumatology, University of California, San Francisco, San Francisco, California, USA; Zuckerberg San Francisco General Hospital, University of California, San Francisco, San Francisco, California, USA; Division of Rheumatology, University of California, San Francisco, San Francisco, California, USA; Division of Hospital Medicine, University of California, San Francisco, San Francisco, California, USA; Division of General Internal Medicine, University of California, San Francisco, San Francisco, California, USA; Division of HIV, Infectious Diseases, and Global Medicine, University of California, San Francisco, San Francisco, California, USA; Division of Infectious Diseases, Department of Medicine, University of California San Francisco, San Francisco, California, USA; San Francisco Department of Public Health Prevention and Control Program, San Francisco, California, USA; Division of Experimental Medicine, Department of Medicine, University of California San Francisco, San Francisco, California, USA

**Keywords:** tuberculosis infection, latent tuberculosis, screening, immunotherapy, guidelines

## Abstract

**Background:**

Targeted immunotherapies have transformed the treatment of many diseases. However, some increase the risk of tuberculosis (TB) disease. We sought to develop a comprehensive list of targeted immunotherapies with TB infection screening recommendations in US Food and Drug Administration (FDA)-approved prescribing information and compare these recommendations to clinical resources and quality measures.

**Methods:**

Through a gray literature review, we identified TB clinical resources and US quality measures. We created a list of targeted immunotherapies and TB infection screening recommendations by analyzing 4 FDA databases. We then evaluated the consistency of screening recommendations in prescribing information, TB clinical resources, and quality measures.

**Results:**

We identified 6 TB clinical resources and 1 quality measure for TB infection. While TB infection screening recommendations for tumor necrosis factor (TNF) inhibitors were consistently included, recommendations for other therapies were less consistent. Through FDA database analyses, we identified 269 targeted immunotherapies, 35 (13%) of which had TB infection screening recommendations in prescribing information, including all therapies targeting TNF and several interleukins (IL); however, therapies targeting IL-6, Janus-associated kinase, and others had variable recommendations. Significant discordance in screening recommendations for immunotherapies was further identified when comparing prescribing information, clinical resources, and quality measures.

**Conclusions:**

The number and targets of immunotherapies are rapidly evolving resulting in challenges with creating up-to-date and consistent TB infection screening recommendations. Inconsistent recommendations in clinical resources may contribute to gaps in TB preventive care. Harmonized recommendations and additional epidemiologic studies of TB disease risk with the use of these agents are needed.

## Teaser

Targeted immunotherapies are increasing in number and complexity but may increase tuberculosis risk, highlighting the challenge of creating tuberculosis infection screening recommendations. We systematically identified these therapies and compared screening recommendations in prescribing information, clinical resources, and quality measures.

There has been rapid growth in the development and use of targeted immunotherapies, including biologics (ie, monoclonal antibodies and fusion proteins) and targeted small molecule inhibitors (eg, kinase inhibitors) that modulate key pathways or cells of the immune response [1]. In some clinical fields, these agents have different names, such as biologic or targeted synthetic disease modifying anti-rheumatic drugs (b/tsDMARDs) in rheumatology. In recent years, these therapies have transformed the treatment of autoimmune disease, cancer, organ transplantation, and other conditions [2–4]. Some therapies, however, also increase the risk of infections, including tuberculosis (TB) [5–11] where individuals may present with more severe or atypical disease due to a diminished or modulated immune response [12, 13]. Although the rate of TB disease in low-incidence settings is <10 per 100 000 persons per year [14], the use of some targeted immunotherapies has been associated with a 5–10-fold higher risk of TB compared to the general population [13, 15]. Importantly, this risk of TB is known to vary by and within class of therapies [16], dose [13], and duration of time on therapy [17].

Despite patient safety concerns and the growing number of approved immunotherapies that may increase TB risk, current US TB clinical guidelines and risk assessment tools vary widely in their recommendations for TB infection screening for different agents [18, 19]. Notable guidelines from outside of the United States include consensus documents from clinical societies in Austria [20], Spain [21], and the European Society of Clinical Microbiology and Infectious Diseases (ESCMID) [22] and expert reviews [1] as well as guidelines from national health systems, such as the United Kingdom's National Health Service [23, 24]. The European guidelines have many similarities (eg, consistent recommendations for screening with tumor necrosis factor [TNF] inhibitor use), but like US guidelines have discordant screening recommendations for several agents and unclear methods for identifying and updating the lists of immunotherapies included.

Clinical practice patterns for TB infection screening with immunotherapies have notably been shown to vary. A recent global survey of TB providers through the Global TB Network highlighted significantly different TB infection screening practices for immunotherapies [25]. Additionally, specialist providers who have familiarity with immunotherapies in their given field have also been found to variably complete recommended TB infection screening for some immunotherapies [26–30]. More broadly, generalists and infectious disease physicians are increasingly taking care of individuals on or previously taking immunotherapies for diverse conditions [31]. The rapid increase in the number and complexity of these therapies can lead to gaps in provider knowledge and lower confidence in prescribing and managing side effects [32, 33]. Inconsistent guidelines on TB infection screening may also contribute to provider uncertainty about best practices, contributing to gaps in TB preventive care [26].

This study had 3 main aims: (1) review US and global TB clinical guidelines and resources, risk assessment tools, and quality measures related to TB infection screening for targeted immunotherapies; (2) create a list of targeted immunotherapies approved in the United States and identify TB infection screening recommendations in Food and Drug Administration (FDA)–approved prescribing information; and (3) compare identified clinical resources and prescribing information to assess the alignment of TB infection screening recommendations.

## METHODS

### Review of Tuberculosis Guidelines, Clinical Resources, Tuberculosis Risk Assessment Tools, and Quality Measures

To identify US and global TB guidelines and clinical resources, our search strategy included a gray literature review using the Grey Matters Lite tool from Canada's Drug Agency [34] as well as a restricted PubMed search [35]. We performed both searches on 22 January 2025. Details of each search can be found in the Supplementary Materials. We identified published risk assessment tools used to estimate the risk of progression of TB infection to disease through a review of identified guidelines as well as discussion among our study team. To identify US clinical quality measures for TB screening, we searched the Centers for Medicare and Medicaid Services’ (CMS) Merit-based Incentive Payment System (MIPS) [36], a component of Medicare's Quality Payment Program, and the Healthcare Effectiveness Data and Information Set (HEDIS), a widely used set of standardized clinical quality measures [37].

### Creation of a Comprehensive List of Targeted Immunotherapies Approved for Use in the United States

The diverse uses of targeted immunotherapies and the rapid pace of approvals are challenges in creating and updating lists of TB infection screening recommendations or general safety checklists for these therapies. To create a comprehensive list of these therapies, we systematically searched 3 FDA databases: the National Drug Code Directory (NDCD) [38], the Approved Drug Products with Therapeutic Equivalence (Orange Book) [39], and the List of Licensed Biological Products with Reference Product Exclusivity and Biosimilarity or Interchangeability Evaluations (Purple Book) [40]. A brief overview of these databases is provided in Table 1 [41]. We downloaded drug lists from all 3 databases on 26 January 2023 and compiled lists of potential immunotherapies from each using 4 steps (Supplementary Figure 1): (1) exclusion of drugs not of interest based on routes of administration (eg, topical or nasal) or product types (eg, over-the-counter); (2) inclusion using pharmaceutical class information or mechanism of action where available; (3) inclusion using US Adopted Name (USAN) suffixes suggestive of targeted immunotherapies (eg, -mab, -ib, -cept) [42] or prefixes for interferons (IFNs); and (4) consolidation and de-duplication of all identified drugs. We updated this list by repeating this process on 4 October 2024.

**Table 1.** Characteristics of 3 US Food and Drug Administration Databases Used to Identify Targeted Immunotherapies With Increased Tuberculosis Risk

	National Drug Code Directory	Orange Book	Purple Book
Overview	Created to maintain a list of all drugs currently marketed in the United States	Created to provide information on therapeutic equivalence to contain drug costs	Created to identify biologics and their biosimilar or interchangeable options; information on product exclusivity
Approval status	FDA-approved and FDA-unapproved drugs	FDA-approved drugs	FDA-approved drugs
Drugs included in database	Finished drugs: prescription and over-the-counter drugs Unfinished drugs Compounded drugs Medical gases Homeopathic drugs	Prescription drugs Over-the-counter drugs	Biologics Vaccines Gene/cell therapies Hematologic products Antibodies
Drugs not included in database	Drugs no longer marketed Drugs solely included in kits or combination products	Drugs discontinued before 1987 Drugs with tentative FDA approval FDA-approved drugs withdrawn for safety or effectiveness reasons Biologics (see Purple Book)	Non-biologic products
Source of data	Drug establishments^a^ Updated: daily	FDA Updated: Daily (generic approvals) Every 2 weeks (new drug approvals)	FDA Updated: every 30 days

Abbreviation: FDA, US Food and Drug Administration.

^a^Drug establishment: any domestic or foreign entity involved in the manufacture, preparation, propagation, compounding, or processing (eg, repackaging and relabeling) of a drug for distribution in the United States or offered for import to the United States is required to register with and submit a list of all drug products to the FDA. Components of this data are published by the FDA through the National Drug Code Directory. The contents of this information are the responsibility of drug establishments and not the FDA, and inclusion does not indicate FDA approval.

After creating the consolidated drug list, we incorporated drug classification and mechanism of action data from DrugBank Online [43]. Study team members with expertise in immunology, infectious diseases, TB clinical care, and pharmacology then collectively reviewed therapies in team meetings and excluded potential therapies for any of the following reasons: non-targeted mechanism of action (eg, alkylating agent, antimetabolite, nucleoside analog), targeted therapy but non-immunologic target (eg, aromatase inhibitor, anti-respiratory syncytial virus antibody), non-systemic route of administration (eg, ophthalmic or intravesical), cellular/gene therapy, or radiopharmaceuticals considered outside of the scope of our analysis.

### Analysis of Food and Drug Administration–Approved Prescribing Information to Identify Immunotherapies With Tuberculosis Infection Screening Recommendations

The FDALabel database [44] allows for the full-text search of FDA-approved prescribing information. This information for providers is a component of overall drug labeling, which also includes information for patients (eg, patient package inserts) and end-users (eg, package labeling). Prescribing information is initially proposed by a drug company and then reviewed and approved by the FDA. The structure of this information is regulated by US federal law and currently contains 17 sections, including indications for use, warnings, precautions, and adverse reactions among others [45]. Drug labeling may be updated when drug companies submit supplemental information to the FDA or the FDA requires the review of new safety information identified passively during post-marketing surveillance through the FDA Adverse Event Reporting System or through proactive safety monitoring via a Risk Evaluation and Mitigation Strategy for agents with safety concerns [46].

We searched the FDALabel database on 4 October 2024 using the search terms tubercul% OR “TB” OR mycobacter%, where “%” represents a wildcard. We compiled a list of drugs with prescribing information containing potential TB-related terms which we manually reviewed for any TB infection screening recommendation or reference to clinical or animal data suggesting an increased risk of TB disease with use. By comparing the list of therapies with screening recommendations or data suggesting an increased risk of TB with the comprehensive list of targeted immunotherapies, we assessed for consistency of TB infection screening recommendations for all therapies with the same immune cell, molecule, or pathway target.

### Comparison of Clinical Resources, Quality Measures, and Prescribing Information

Lastly, we compared therapies with TB infection screening recommendations in FDA-approved prescribing information with those identified in US and global TB guidelines, clinical resources, risk assessment tools, and quality measures. Among therapies with screening recommendations in prescribing information, we used multivariable logistic regression to evaluate for associations between inclusion in the most recently updated TB clinical guideline [19] and quality measure [36] with therapy indication and number of years since FDA approval. We performed all analyses using Stata version 17 (StataCorp LLC, College Station, TX, USA) and Microsoft Excel (Microsoft, Redmond, WA, USA).

## RESULTS

### Review of Tuberculosis Guidelines, Clinical Resources, Tuberculosis Risk Assessment Tools, and Quality Measures

Our gray literature review and PubMed search identified 13 US and global TB clinical guidelines and resources. Seven of these were excluded due to TB infection screening recommendations being outside guideline scope (*n* = 2) [47, 48], publication > 10 years ago (*n* = 1) [49], newer version of guideline available (*n* = 3) [50, 51, 52], and target audience of providers in high-TB-incidence settings (*n* = 1) [53]. All 6 included TB guidelines and clinical resources published since 2020 consistently recommended screening for TB infection with TNF inhibitor use [18, 19, 54–57]. Recommendations ranged from TB infection screening with the use of all targeted immunotherapies [18] to certain classes of therapies [19 , 54] with one guideline advising providers to review manufacturer drug labels or consult a TB specialist if needed [19]. The 2 most recently updated US guidelines and resources [19, 54] included consistent screening recommendations for agents targeting cluster of differentiation (CD)-20; cytotoxic T-lymphocyte–associated antigen (CTLA)-4; interleukin (IL)-6, IL-12/IL-23, IL-17, and IL-23; and Janus-associated kinase (JAK) inhibitors. Differences between these resources existed for therapies targeting B-lymphocyte stimulator protein (BLyS), IL-1, and type II IFN (ie, IFN gamma) (Table 2).

**Table 2.** Comparison of Targeted Immunotherapies With Tuberculosis (TB) Infection Screening Recommendations in FDA-Approved Prescribing Information, US and Global TB Guidelines and Clinical Resources, Risk Assessment Tools, and Clinical Quality Measures

Resource		Year	Immunotherapy: Targeted Cell, Molecule, or Pathway							
			TNF	BLyS	CD-19	CD-20	CD-52	CTLA-4	Type I IFN	Type 2 IFN (IFN-Gamma)
Current analysis: targeted immunotherapies with TB infection screening recommendation in FDA-approved prescribing information										
FDA prescribing information	-	2025	adalimumab certolizumab etanercept golimumab infliximab	-	inebilizumab	-	alemtuzumab	abatacept belatacept	-	emapalumab
US guidelines/clinical resources										
Publisher										
NTCA/NSTC	Testing and treatment of latent tuberculosis infection in the United States: clinical recommendations	2024	adalimumab certolizumab etanercept golimumab infliximab	belimumab	-	rituximab	-	abatacept	-	-
UpToDate	Risk of mycobacterial infection associated with biologic agents and JAK inhibitors	2025	adalimumab certolizumab etanercept golimumab infliximab	-	-	rituximab	-	abatacept	anifrolumab	-
AAP	Tuberculosis infection in children and adolescents: testing and treatment	2021	TNF inhibitor	Explicitly mentions TNF inhibitor, noting people who are “receiving immunosuppressive therapy, such as TNF-alpha antagonists, systemic corticosteroids equivalent to/greater than 15 mg of prednisone per day, or immunosuppressive drug therapy following organ transplantation” are at high risk of developing TB disease after TB infection						
CDC	Latent tuberculosis infection: a guide for primary health care providers	2020	TNF inhibitor	Explicitly mentions TNF inhibitors but also highlights that “it is commonly recommended that all patients who will be receiving an immunomodulating biological agent, regardless of specific TB risk factors, should be tested for TBI before starting the therapy”						
Global guidelines/clinical resources										
WHO	WHO consolidated guidelines on tuberculosis: tuberculosis preventive treatment	2024	TNF inhibitor	-	-	-	-	-	-	-
IJTLD Clinical Standards for TB Infection	Clinical standards for the diagnosis, treatment and prevention of TB infection	2022	TNF inhibitor	-	-	-	-	-	-	-
Risk assessment/clinical decision support tools										
CDPH	California tuberculosis risk assessment	2024	TNF inhibitor	TB infection testing is recommended for any of the following: current or planned immunosuppression, organ transplant recipient, treated with biologic agents including TNF antagonist (eg, infliximab, adalimumab, etanercept, others), steroids (equivalent of prednisone ≥15 mg/day for ≥1 m), or other immunosuppressive medication						
LTBI-ASSIST	Latent TB infection ASSIST: interactive decision support for current CDC TB guidelines	2022	adalimumab certolizumab etanercept golimumab infliximab	-	-	rituximab	-	abatacept	-	-
Clinical quality measures										
US CMS: Merit-based Incentive Payment Scheme	Measure #176: tuberculosis screening prior to first course of biologic and/or immune response modifier therapy	2025	adalimumab certolizumab etanercept golimumab infliximab	-	-	-	-	abatacept	-	-

Resource		Year	Immunotherapy: Targeted Cell, Molecule, or Pathway							
			IL-1	IL-6	IL-12/IL-23	IL-17	IL-23	IL-36	Kinase (JAK)	Integrin Alpha-4
Current analysis: targeted immunotherapies with TB infection screening recommendation in FDA-approved prescribing information										
FDA prescribing information	--	2025	anakinra canakinumab rilonacept	sarilumab satralizumab tocilizumab	ustekinumab	bimekizumab brodalumab ixekizumab secukinumab	guselkumab mirikizumab risankizumab tildrakizumab	spesolimab	abrocitinib baricitinib deucravacitinib deuruxolitinib ritlecitinib ruxolitinib tofacitinib upadacitinib	vedolizumab
US guidelines/clinical resources										
Publisher										
NTCA/NSTC	Testing and treatment of latent tuberculosis infection in the United States: clinical recommendations	2024	anakinra canakinumab rilonacept	sarilumab tocilizumab	ustekinumab	brodalumab ixekizumab secukinumab	guselkumab risankizumab tildrakizumab	-	baricitinib filgotinib peficitinib tofacitinib upadacitinib	-
UpToDate	Risk of mycobacterial infection associated with biologic agents and JAK inhibitors	2025	-	sarilumab tocilizumab	ustekinumab	“IL-17 blockers”	“IL-23 blockers”	-	baricitinib deucravacitinib tofacitinib upadacitinib “And others”	-
AAP	Tuberculosis infection in children and adolescents: testing and treatment	2021	Explicitly mentions TNF inhibitor, noting people who are “receiving immunosuppressive therapy, such as TNF-alpha antagonists, systemic corticosteroids equivalent to/greater than 15 mg of prednisone per day, or immunosuppressive drug therapy following organ transplantation” are at high risk of developing TB disease after TB infection							
CDC	Latent tuberculosis infection: a guide for primary health care providers	2020	Explicitly mentions TNF inhibitors but also highlights that “it is commonly recommended that all patients who will be receiving an immunomodulating biological agent, regardless of specific TB risk factors, should be tested for TBI before starting the therapy”							
Global guidelines/clinical resources										
WHO	WHO consolidated guidelines on tuberculosis: tuberculosis preventive treatment	2024	-	-	-	-	-	-	-	-
IJTLD Clinical Standards for TB Infection	Clinical standards for the diagnosis, treatment and prevention of TB infection	2022	-	-	-	-	-	-	-	-
Risk assessment/clinical decision support tools										
CDPH	California tuberculosis risk assessment	2024	TB infection testing is recommended for any of the following: current or planned immunosuppression, organ transplant recipient, treated with biologic agents including TNF antagonist (eg, infliximab, adalimumab, etanercept, others), steroids (equivalent of prednisone ≥15 mg/day for ≥1 m), or other immunosuppressive medication							
LTBI-ASSIST	Latent TB infection ASSIST: interactive decision support for current CDC TB guidelines	2022	anakinra canakinumab	sarilumab	ustekinumab	brodalumab ixekizumab secukinumab	guselkumab risankizumab tildrakizumab	-	baricitinib tofacitinib upadacitinib	-
Clinical quality measures										
US CMS: Merit-based Incentive Payment Scheme	Measure #176: tuberculosis Screening prior to first course of biologic and/or immune response modifier therapy	2025	anakinra canakinumab	sarilumab tocilizumab	ustekinumab	brodalumab ixekizumab secukinumab	guselkumab risankizumab tildrakizumab	-	baricitinib tofacitinib upadacitinib	-

Abbreviations (guideline/clinical resources): AAP, American Academy of Pediatrics; CDC, Centers for Disease Control and Prevention (United States); CDPH, California Department of Public Health; CMS, Centers for Medicare and Medicaid Services; FDA, US Food and Drug Administration; IDSA, Infectious Diseases Society of America; IJTLD, International Journal of Tuberculosis and Lung Disease; LTBI, latent tuberculosis infection; NTCA/NSTC, National Tuberculosis Coalition of America/National Society of Tuberculosis Clinicians; US, United States; WHO, World Health Organization.

Abbreviations (immune cell, molecule, or pathway targeted of identified therapies): BLyS, B lymphocyte stimulator protein; CD, cluster of differentiation; CTLA, cytotoxic T-lymphocyte–associated antigen; IFN, interferon; IL, interleukin; JAK, Janus-associated kinase, includes tyrosine kinase 2 (TYK2); TNF, tumor necrosis factor.

ATS/CDC/IDSA guidelines (2017): immunosuppressive therapy included in paradigm for evaluation of those with latent tuberculosis infection as a factor that increases risk of developing tuberculosis if infected. No other mention in text of specific therapies [47].

USPSTF guidelines (2023): focus on non-US born persons and persons who have lived in high-risk congregate settings. Although guidelines note that TNF inhibitors increase risk of TB disease, USPSTF did not include recommendations on TB infection screening for immunosuppressive therapies [48].

Two risk assessment and clinical decision support tools from the United States were identified [58, 59]. Both included recommendations for TB infection screening for all current or planned immunosuppressive therapy with a specific list of therapies in LTBI-ASSIST based on a prior version of the National Tuberculosis Coalition of America (NTCA) guidelines (Table 2) [60]. Widely cited tools from Canada and the United Kingdom to estimate risk of progression from TB infection to disease include general risk factors of cancer, transplant [61], and less commonly TNF inhibitor use [62]. One quality measure for TB infection screening in the United States was identified from CMS's MIPS, but none from HEDIS (Table 2) [63]. Immunotherapies included in the MIPS measure [64] have evolved significantly over time, where separate measures were developed and maintained by the American College of Dermatology for psoriasis, psoriatic arthritis, and rheumatoid arthritis and another by the American College of Rheumatology for rheumatoid arthritis until 2020 and then all diagnoses in 2021. These measures were combined into the current measure in 2022 (Supplementary Table 1) [36].

### Creation of a Comprehensive List of Targeted Immunotherapies Approved for Use in the United States

We identified 497 potential targeted immunotherapies from 3 FDA databases. Through study team review, 228 (46% of 497) were excluded: 83 (36% of 228) non-targeted therapies (eg, alkylating agents), 111 (49%) targeted therapies without an immunologic target (eg, anti-amyloid beta antibody), 25 (11%) cellular and gene therapies not considered within the scope of this analysis, 7 (3%) therapies not administered systemically, and 2 (<1%) radiopharmaceuticals (Supplementary Figure 1 and Table 2). In total, 269 targeted immunotherapies were identified for further review.

### Analysis of Food and Drug Administration–Approved Prescribing Information to Identify Immunotherapies With Tuberculosis Infection Screening Recommendations

Of the 269 identified targeted immunotherapies, the FDA-approved prescribing information for 35 (13%) included a recommendation for TB infection screening. These therapies target TNF, CTLA-4, CD-19, CD-52, type II IFN, IL-1, IL-6, IL-12/23, IL-17, IL-23, IL-36, integrin alpha-4, JAK, and tyrosine kinase 2 (TYK2) (Table 3). The exact wording of identified recommendations is included in Supplementary Table 3. The prescribing information for an additional 25 immunotherapies (9% of 269) included text noting a possible risk of TB disease based on animal data only (*n* = 10 inhibitors of programmed cell death protein-1 [PD-1] and programmed death ligand-1 [PD-L1]), clinical data (*n* = 13 total, including 2 mammalian target of rapamycin [mTOR] inhibitors and 3 nuclear factor-erythroid factor 2-related factor 2 [Nrf2] activators), or general notes of caution without a screening recommendation (*n* = 2) (Supplementary Table 4). Tuberculosis infection screening recommendations in prescribing information were identified for all immunotherapies suppressing CTLA-4 and targeting CD-52, IL-1, IL-12/IL-23, IL-17, IL-23, IL-36, TNF, and type II IFN. Similarly, the prescribing information for all PD-1/PD-L1 inhibitors included a statement about possible increased TB risk without a screening recommendation. The presence of a screening recommendation was not consistent for several immunotherapies with the same immunologic target, including CD-19, integrin alpha-4, IL-6, and JAK (Table 4; Supplementary Table 5).

**Table 3.** Targeted Immunotherapies With Tuberculosis Infection Screening Recommendations in Prescribing Information

Nonproprietary Drug Name (n = 35)	Targeted Cell, Molecule, or Pathway	Proprietary Drug Name(s): Reference Product, Biosimilar and Interchangeable	Earliest FDA Approval Date for Drug	Indications					
				Rheumatology	Hematology/Oncology	Gastroenterology	Neurology	Dermatology	Infectious Disease/Transplant
adalimumab	TNF	Abrilada, Amjevita, Cyltezo, Hadlima, Hulio, Humira, Hyrimoz, Idacio, Simlandi, Yuflyma, Yusimry	12/31/02	RA, PJIA, PsA, AS, uveitis		CD, UC		PsO, HS	
certolizumab	TNF	Cimzia	4/22/08	RA, PJIA, PsA, AS, non-radiographic AS		CD		PsO	
golimumab	TNF	Simponi	4/24/09	RA, PsA, AS, PJIA		UC			
infliximab	TNF	Avsola, Inflectra, Ixifi, Remicade, Renflexis, Zymfentra	8/24/98	RA, AS, PsA		CD, UC		PsO	
etanercept	TNF	Enbrel, Erelzi, Eticovo	11/2/98	RA, PJIA, PsA, juvenile PsA, AS				PsO	
abatacept	CTLA-4	Orencia	12/23/05	RA, PJIA, PsA	Prophylaxis for acute GVHD				
belatacept	CTLA-4	Nulojix	6/15/11						Adult kidney transplant recipients
inebilizumab	CD-19	Uplizna	6/11/20				NMOSD		
alemtuzumab	CD-52	Campath, Lemtrada	5/7/01		B-cell CLL		MS		
emapalumab	Type II IFN (IFN-gamma)	Gamifant	11/20/18		Primary HLH				
anakinra	IL-1	Kineret	11/14/01	RA CAPS, DIRA					
canakinumab	IL-1	Ilaris	6/17/09	Gout CAPS (including FCAS, MWS), TRAPS, HIDS/MKD, FMF Still's Disease: AOSD, SJIA					
rilonacept	IL-1	Arcalyst	2/27/08	CAPS (including FCAS, MWS), DIRA Pericarditis (recurrent)					
sarilumab	IL-6	Kevzara	5/22/17	RA, PMR, PJIA					
satralizumab	IL-6	Enspryng	8/14/20				NMOSD		
tocilizumab	IL-6	Actemra, Tofidence, Tyenne	1/8/10	RA, PJIA, SJIA GCA, SSc-ILD, CRS					COVID-19
ustekinumab	IL-12/IL-23	Imuldosa, Otulfi, Pyzchiva, Selarsdi, Stelara, Wezlana	9/25/09	PsA		CD, UC		PsO	
bimekizumab	IL-17	Bimzelx	10/17/23	PsA, AS, non-radiographic AS				PsO	
ixekizumab	IL-17	Taltz	3/22/16	PsA, AS, non-radiographic AS				PsO	
secukinumab	IL-17	Cosentyx	1/21/15	PsA, AS, non-radiographic AS, enthesitis-related arthritis				PsO, HS	
brodalumab	IL-17	Siliq	2/15/17					PsO	
guselkumab	IL-23	Tremfya	7/13/17	PsA		UC		PsO	
mirikizumab	IL-23	Omvoh	10/26/23			UC			
risankizumab	IL-23	Skyrizi	4/23/19	PsA		CD, UC		PsO	
tildrakizumab	IL-23	Ilumya	3/20/18					PsO	
spesolimab	IL-36	Spevigo	9/1/22					GPP	
vedolizumab	Integrin (α4β7)	Entyvio	5/20/14			CD, UC			
abrocitinib	Kinase inhibitor (JAK1)	Cibinqo	1/14/22					AD	
baricitinib	Kinase inhibitor (JAK1/JAK2)	Olumiant	5/31/18	RA				AA	COVID-19
deuruxolitinib	Kinase inhibitor (JAK1/JAK2)	Leqselvi	7/25/24					AA	
ritlecitinib	Kinase inhibitor (JAK3)	Litfulo	6/23/23					AA	
ruxolitinib	Kinase inhibitor (JAK1/JAK2)	Jakafi, Jakavi, Opzelura	11/16/11		Myelofibrosis, PV, acute GVHD, chronic GVHD			AD, vitiligo	
tofacitinib	Kinase inhibitor (JAK1/JAK2/JAK3)	Xeljanz	11/6/12	RA, PsA, AS, PJIA		UC			
upadacitinib	Kinase inhibitor (JAK1)	Rinvoq	8/16/19	RA, PsA, AS, non-radiographic AS, PJIA		CD, UC		AD	
deucravacitinib	Kinase inhibitor (TYK2)	Sotyktu	9/9/22					PsO	

Abbreviation (general): FDA, US Food and Drug Administration.

Abbreviations (immune cell, molecule, or pathway targeted of identified therapies): CD, cluster of differentiation; CTLA, cytotoxic T-lymphocyte-associated antigen; IFN, interferon; IL, interleukin; JAK, Janus kinase; TNF, tumor necrosis factor; TYK, tyrosine kinase.

Abbreviations (indications): Rheumatology—CAPS, cryopyrin-associated periodic syndromes, including FCAS, familial cold autoinflammatory syndrome; MWS, Muckle–Wells syndrome; DIRA, deficiency of interleukin-1 receptor antagonist; TRAPS, tumor necrosis factor receptor associated periodic syndrome; HIDS, hyperimmunoglobulin D syndrome; MKD, mevalonate kinase deficiency; FMF, familial Mediterranean fever; CRS, cytokine release syndrome; AOSD, adult-onset Still's disease; SJIA, systemic juvenile idiopathic arthritis; RA, rheumatoid arthritis; PJIA, polyarticular juvenile idiopathic arthritis; AS, ankylosing spondylitis; PsA, psoriatic arthritis; PMR, polymyalgia rheumatica; GCA, giant cell arteritis; SSc-ILD, systemic sclerosis-associated interstitial lung disease. Hematology/oncology—CLL, chronic lymphocytic leukemia; GVHD, graft-vs-host disease; HLH, hemophagocytic lymphohistiocytosis; PV, polycythemia vera. Gastroenterology—CD, Crohn's disease; UC, ulcerative colitis. Neurology—MS, multiple sclerosis; NMOSD, neuromyelitis optica spectrum disorder. Dermatology—AA, alopecia areata; AD, atopic dermatitis; HS, hidradenitis suppurativa; PsO, psoriasis. Infectious disease/transplant—COVID-19, coronavirus disease 2019.

**Table 4.** Concordance and Discordance of Tuberculosis Infection Screening Recommendations in Prescribing Information for Immunotherapies by Targeted Immune Cell, Molecule, or Pathway

Category of Immunotherapy	Targeted Cell, Molecule, or Pathway of Immunotherapy	Recommendation for Tuberculosis Infection Screening	Possible Increased Tuberculosis Risk: Statement, Clinical or Animal Data	No Screening Recommendation, Data or Statement on Possible Increased Tuberculosis Risk
Concordant prescribing information: tuberculosis (TB) infection screening recommendation for all immunotherapies with same molecular/cellular target				
T cell	CTLA-4 (suppression)	abatacept, belatacept		
B and T cells	CD-52	alemtuzumab		
Interferon	Type II interferon	emapalumab		
Interleukin	IL-1	anakinra, canakinumab, rilonacept		
Interleukin	IL-12/IL-23	ustekinumab		
Interleukin	IL-17	bimekizumab, brodalumab, ixekizumab, secukinumab		
Interleukin	IL-23	guselkumab, mirikizumab, risankizumab, tildrakizumab		
Interleukin	IL-36	spesolimab		
TNF	TNF	adalimumab, certolizumab, etanercept, golimumab, infliximab		
Concordant prescribing information: statement/data suggesting possible increased TB risk for all immunotherapies with same molecular/cellular target				
Complement	C3		pegcetacoplan	
Immune checkpoint^a^	PD-1/PD-L1		atezolizumab, avelumab, cemiplimab, dostarlimab, durvalumab, nivolumab, nivolumab and relatlimab, pembrolizumab, retifanlimab-dlwr, tislelizumab, toripalimab	
Discordant prescribing information: subset of immunotherapies with same molecular/cellular target stating possible increased risk for TB disease with use				
B and T cells	CD-38		isatuximab	daratumumab
Cell surface receptors and pathways	VEGF/EGFR/Erb		erlotinib	amivantamab, cetuximab, necitumumab, panitumumab, dacomitinib, lazertinib, mobocertinib, lapatinib, neratinib, afatinib, gefitinib, osimertinib, nintedanib, cabozantinib, pazopanib, regorafenib, sorafenib, sunitinib, vandetanib, axitinib, lenvatinib, tivozanib, ramucirumab, bevacizumab, ranibizumab, aflibercept, fruquintinib, ziv-aflibercept
Complement	C5a		avacopan	vilobelimab
Interferon	Type I interferon		peginterferon alfa-2a	interferon alfa-2a, interferon alfa-2b, interferon alfa-n3 (human leukocyte derived), interferon alfacon-1, interferon beta-1a, interferon beta-1b, interferon gamma-1b, peginterferon alfa-2a and ribavirin, peginterferon alfa-2b, peginterferon beta-1a, ropeginterferon alfa-2b
Intracellular signaling	Kinase (ALK)		ceritinib	alectinib, brigatinib, crizotinib, lorlatinib
Intracellular signaling	mTOR		everolimus, sirolimus	temsirolimus
Other	Nrf2 signaling		dimethyl fumarate, diroximel fumarate, monomethyl fumarate	omaveloxolone
Other	Proteasome		carfilzomib	bortezomib, ixazomib
Other	Sphingosine 1-phosphate		fingolimod	etrasimod, ozanimod, ponesimod, siponimod
Discordant prescribing information: subset of immunotherapies with same molecular/cellular target with recommendation for TB infection screening with use				
B cell	CD-19	inebilizumab		blinatumomab, tafasitamab-cxix, loncastuximab tesirine
Adhesion molecules	Integrin alpha-4	vedolizumab		natalizumab
Interleukin	IL-6	sarilumab, satralizumab, tocilizumab		siltuximab
Intracellular signaling	Kinase (JAK/STAT)	abrocitinib, upadacitinib, baricitinib, deuruxolitinib, ruxolitinib, tofacitinib, ritlecitinib, deucravacitinib	pacritinib	momelotinib, fedratinib

Abbreviations (immune cell, molecule, or pathway targeted of identified therapies): ALK, anaplastic lymphoma kinase; CD, cluster of differentiation; CTLA, cytotoxic T-lymphocyte-associated antigen; EGFR, epidermal growth factor receptor; FLT, FMS-like tyrosine kinase; IL, interleukin; JAK, Janus kinase; mTOR, mammalian target of rapamycin; Nrf2, nuclear factor-erythroid factor 2-related factor 2; PD-1, programmed cell death protein 1; PD-L1, programmed death-ligand 1; STAT, signal transducer and activator of transcription; TNF, tumor necrosis factor; VEGF, vascular endothelial growth factor.

^a^Immune checkpoint inhibitors targeting CTLA-4 (ipilimumab, tremelimumab) do not have recommendations for TB infection screening or statements/data of possible increased TB risk in their prescribing information.

### Comparison of Clinical Resources, Quality Measures, and Prescribing Information

Significant discordance in TB infection screening recommendations for immunotherapies was identified across prescribing information, clinical resources, risk assessment tools, and quality measures. As several identified resources recommended consideration of TB infection screening with all immunosuppressive therapies [18, 55, 58], we focused our analysis of recommendation agreement on prescribing information, the 2025 NTCA TB infection guidelines [19], UpToDate [54], and the 2025 CMS quality measure MIPS #176 (Supplementary Table 6) [64]. For these resources, only the screening recommendations for immunotherapies targeting TNF (*n* = 5) and IL-12/IL-23 inhibitors (*n* = 1, ustekinumab) were in complete agreement. Five therapies had specific TB infection screening recommendations in clinical resources but not in prescribing information: belimumab, rituximab, anifrolumab, and 2 JAK inhibitors included in the NTCA guidelines not approved for use in the United States (filgotinib, peficitinib). Of the 35 identified immunotherapies with TB infection screening recommendations in prescribing information, 14 were not included in NTCA guidelines (40%) [19], 10 were not included in the UpToDate article reviewed (29%) [54], and 15 were not included in the CMS quality measure (43%) [64]. In a multivariable logistic regression model, inclusion of an immunotherapy in the CMS quality measure was highly associated with an indication for rheumatoid arthritis, psoriasis, or psoriatic arthritis (19/22 therapies with an indication were included versus 1/13 therapies without an indication; adjusted odds ratio [aOR] 3645, 95% CI 4.45–2 987 587, *P* = .017) and increasing number of years since FDA approval (aOR 1.56 per year, 95% CI 1.03–2.36, *P* = .036). Findings were similar for a separate model with inclusion in NTCA guidelines as the outcome.

## DISCUSSION

Targeted immunotherapies are rapidly evolving, highlighting the challenge of creating and updating TB infection screening recommendations for these agents. By leveraging multiple complementary FDA databases, we established a reproducible approach to comprehensively identify targeted immunotherapies approved for use in the United States and assess for TB infection screening recommendations included in prescribing information. Using a gray literature review, we identified TB guidelines and clinical resources, TB risk assessment tools, and quality measures and then compared screening recommendations in these resources to FDA-approved prescribing information. Despite consistency of recommendations with the use of immunotherapies targeting TNF and IL-12/23, many therapies with screening recommendations in prescribing information were not included in clinical resources or the CMS quality measure. Notably, all identified therapies targeting CD-19, CD-52, IL-36, integrin alpha-4, and type II IFN were absent, and there were discrepancies for therapies targeting CTLA-4, several interleukins (ie, IL-1, IL-6, IL-17, IL-23), and JAK. Examining only prescribing information, immunotherapies targeting CD-19, integrin alpha-4, IL-6, and JAK also did not have consistent recommendations.

Some immunotherapies without TB infection screening recommendations in clinical resources or prescribing information, such as PD-1/PD-L1 inhibitors, have been highlighted as having possible increased risk of TB disease in recent systematic reviews [8, 65, 66]. Depending on the class of therapy or specific agent, discordant screening recommendations may be due to a combination of factors, including actual TB disease risk, different levels of evidence, or a lack of data to inform recommendations and delays in updating recommendations or lists of immunotherapies. Despite safety evidence in low-TB-incidence settings, TB infection screening for some agents may be recommended due to a theoretical risk for TB disease or the provision of TB infection treatment in key clinical trials (eg, IL-17 inhibitors) [67, 68]. Given the pace of immunotherapy research and approvals, updates to FDA-approved prescribing information may also lag behind safety evidence. This lag has been shown generally for indications of drugs [69] as well as safety warnings [70]. Pharmaceutical companies may also be disincentivized from submitting supplements due to the cost and time involved in applications [71].

The evidence for TB infection screening is strongest for TNF inhibitors but may be inconclusive for other therapies due to the recency of regulatory approval, lack of studies, clinical trials excluding individuals with TB infection, or other reasons. For post-marketing studies, immunotherapy exposure is also complex and time variable [72], often involving medication changes [73]. Ascertainment of TB disease outcomes can be complicated by fragmented public health and clinical data systems requiring linkage. Several key confounders of the relationship between immunotherapies and TB disease also must be considered, including underlying medical conditions, medication, and TB history. Large observational studies integrating disparate clinical and claims data [74] as well as systematic reviews are necessary to strengthen the evidence base for TB infection screening recommendations for immunotherapies.

One of the key strengths of this work was the systematic approach of identifying targeted immunotherapies and TB infection screening recommendations with the potential to rapidly update these analyses to account for the evolving landscape of these therapies, changes to prescribing information or newly identified adverse effects. This work also had key limitations. We focused on immunotherapies approved for use in the United States and not those available globally. Despite an interdisciplinary study team review, it is possible that some therapies were misclassified as immunotherapy or incorrectly excluded. Our literature search strategy did not include subspecialty guidelines, which may include screening recommendations. The findings of this analysis reflect the screening recommendations at the time of FDA analysis and clinical resource review. Lastly, we did not analyze the quality of evidence underpinning the presence or absence of recommendations.

Clinically, decisions to screen for and treat TB infection prior to the use of immunotherapies are often more complex than a binary recommendation. In addition to discordant recommendations, providers must weigh the local prevalence of TB infection and TB disease [75] and that individuals seeking care may have multiple risk factors for TB infection or progression to TB disease [23], a prior history of TB disease [76], significant drug–drug interactions with TB preventive therapies, or a clinical urgency to start immunotherapy. Interferon-gamma release assays and tuberculin skin tests are useful but imperfect tests of immunoreactivity and not infection [77] and as a result can be impacted by medical conditions and therapies that modulate the immune system [78, 79]. Consequently, false-negative TB infection tests can occur depending on the timing of screening in relation to targeted immunotherapy or other immunosuppressive medication use as well as the degree of immune system modulation by underlying medical conditions [78]. In order to support providers, there is a need to create transparent strategies to update lists of immunotherapies, consolidate and harmonize recommendations across clinical resources, and develop and implement tools for reducing gaps in screening for immunotherapies with the highest risk of TB [80, 81].

## Supplementary Material

ciaf628_Supplementary_Data

## Supplementary Data

Supplementary materials are available at *Clinical Infectious Diseases* online. Consisting of data provided by the authors to benefit the reader, the posted materials are not copyedited and are the sole responsibility of the authors, so questions or comments should be addressed to the corresponding author.
