# Supplementary material for: Tuberculosis Infection Screening Recommendations for Targeted Immunotherapies: Comparison of US Prescribing Information, Clinical Resources, and Quality Measures
Source: Clin Infect Dis. 2025 Nov 19;82(5):e1023–35. doi: 10.1093/cid/ciaf628 (PMC13189675; doi:10.1093/cid/ciaf628)
Supplement: ciaf628_Supplementary_Data [file ciaf628_supplementary_data.zip › Supplemental Figure 1 - 2025Jun25.docx]

**Supplemental Figure 1:** Identification of targeted immunotherapies in three U.S. Food and Drug Administration databases.

**ALT TEXT:** flowchart showing the eligibility of drugs identified in three U.S. Food and Drug Administration databases to create a comprehensive list of targeted immunotherapies approved for use in the U.S.
