# Supplementary material for: Tuberculosis Infection Screening Recommendations for Targeted Immunotherapies: Comparison of US Prescribing Information, Clinical Resources, and Quality Measures
Source: Clin Infect Dis. 2025 Nov 19;82(5):e1023–35. doi: 10.1093/cid/ciaf628 (PMC13189675; doi:10.1093/cid/ciaf628)
Supplement: ciaf628_Supplementary_Data [file ciaf628_supplementary_data.zip › Supplemental Materials - References - 2025Nov4.docx]

**Supplemental Materials for Tuberculosis infection screening recommendations for targeted immunotherapies: comparison of U.S. prescribing information, clinical resources and quality measures**

**Additional References:**

1. World Health Organization. WHO operational handbook on tuberculosis. Module 1: prevention - infection prevention and control. Geneva: World Health Organization; 2023. Licence: CC BY-NC-SA 3.0 IGO.
2. Latent tuberculosis infection: updated and consolidated guidelines for programmatic management. Geneva: World Health Organization; 2018. Licence: CC BY-NC-SA 3.0 IGO.
3. Dlodlo RA, Brigden G, Heldal E, et al. Management of tuberculosis: a guide to essential practice. Paris, France: International Union Against Tuberculosis and Lung Disease, 2019.
4. Winthrop KL. Risk of mycobacterial infection associated with biologic agents and JAK inhibitors. In: UpToDate, Connor RF (Ed), Wolters Kluwer. July 31, 2023 (Accessed on June 30, 2025).
5. US Centers for Disease Control and Prevention. Latent tuberculosis infection: a guide for primary health care providers. CDC primary care providers, US Department of Health and Human Services. Atlanta, USA: 2020.
6. World Health Organization. consolidated guidelines on tuberculosis. Module 1: prevention – tuberculosis preventive treatment, second edition. Geneva: World Health Organization; 2024. Licence: CC BY-NC-SA 3.0 IGO.
7. Migliori GB, Wu SJ, Matteelli A, et al. Clinical standards for the diagnosis, treatment and prevention of TB infection. International Journal of Tuberculosis and Lung Disease **2022**; 26:190–205.
8. California Department of Public Health. Tuberculosis risk assessment. August 2024. <https://www.cdph.ca.gov/Programs/CID/DCDC/Pages/TB-Risk-Assessment.aspx>
9. Starke SJ, Martinez Rivera MB, Krishnan S, Shah M. Randomized controlled trial of clinical guidelines versus interactive decision-support for improving medical trainees’ confidence with latent tuberculosis care. Journal of General Internal Medicine **2024**; 39:951–959.
10. National Tuberculosis Coalition of America. Testing and treatment of latent tuberculosis infection in the United States: clinical recommendations – a guide for health care providers and public health programs. **2021**.
11. Gupta RK, Calderwood CJ, Yavlinsky A, Krutikov M, Quartagno M, Aichelburg MC, *et al.* Discovery and validation of a personalized risk predictor for incident tuberculosis in low transmission settings. Nature Medicine **2020**; 26:1941–1949.
12. The Online TST/IGRA Interpreter. Version 4.0. McGill University. <https://www.tstin3d.com/calc.html>.
13. Murrill MT, Salcedo K, Tschampl CA, et al. Policy impediments to tuberculosis elimination: consequences of an absent Medicare national coverage determination for tuberculosis prevention. Journal of Immigrant and Minority Health **2025**; 27:403–408.
14. Centers for Medicare and Medicaid Services. Quality Payment Program, Merit-based Incentive Payment System (MIPS). 2025 MIPS Clinical Quality Measures. Quality ID #176: tuberculosis screening prior to first course of biologic and/or immune response modifier therapy. December 2024. <https://qpp.cms.gov/docs/QPP_quality_measure_specifications/CQM-Measures/2025_Measure_176_MIPSCQM.pdf>
15. Dantas LA, Pereira MS, Gauza A de M, et al. Latent tuberculosis infection reactivation in patients with multiple sclerosis in use of disease-modifying therapies: A systematic review. Multiple Sclerosis and Related Disorders **2021**; 55:103184.
16. Zaemes J, Kim C. Immune checkpoint inhibitor use and tuberculosis: a systematic review of the literature. European Journal of Cancer **2020**; 132:168–175.
17. Elewski BE, Baddley JW, Deodhar AA, et al. Association of secukinumab treatment with tuberculosis reactivation in patients with psoriasis, psoriatic arthritis, or ankylosing spondylitis. Journal of the American Medical Association Dermatology **2021**; 157:43–51.
18. Winthrop KL, Mariette X, Silva JT, et al. ESCMID Study Group for Infections in Compromised Hosts (ESGICH) Consensus Document on the safety of targeted and biological therapies: an infectious diseases perspective (Soluble immune effector molecules [II]: agents targeting interleukins, immunoglobulins and complement factors). Clinical Microbiology and Infection **2018**; 24:S21–S40.
19. Shea MB, Stewart M, Van Dyke H, Ostermann L, Allen J, Sigal E. Outdated prescription drug labeling: how FDA-approved prescribing information lags behind real-world clinical practice. Therapeutic Innovation & Regulatory Science **2018**; 52:771–777.
20. Frank C, Himmelstein DU, Woolhandler S, et al. Era of faster FDA drug approval has also seen increased black-box warnings and market withdrawals. Health Affairs **2014**; 33:1453–1459.
21. Balogh EP, Bindman AB, Eckhardt SG, et al. Challenges and opportunities to updating prescribing information for longstanding oncology drugs. Oncologist **2020**; 25:e405–e411.
22. Stricker BHCh, Stijnen T. Analysis of individual drug use as a time-varying determinant of exposure in prospective population-based cohort studies. European Journal of Epidemiol **2010**; 25:245–251.
23. Fletcher A, Lassere M, March L, et al. Patterns of biologic and targeted-synthetic disease-modifying antirheumatic drug use in rheumatoid arthritis in Australia. Rheumatology (Oxford, England) **2022**; 61:3939.
24. Yan M, Hernandez A, Chan KK, et al. Population-based analysis of the risk of mycobacterial infections associated with immune checkpoint inhibitors. Clinical Infectious Diseases **2025**; 81(1):176-178.
25. Kedia S, Mouli VP, Kamat N, et al. Risk of tuberculosis in patients with inflammatory bowel disease on infliximab or adalimumab is dependent on the local disease burden of tuberculosis: a systematic review and meta-analysis. American Journal of Gastroenterology **2020**; 115:340–349.
26. Brehm TT, Reimann M, Köhler N, Lange C. (Re-)introduction of TNF antagonists and JAK inhibitors in patients with previous tuberculosis: a systematic review. Clinical Microbiology and Infection **2024**; 30:989–998.
27. Emery JC, Richards AS, Dale KD, et al. Self-clearance of Mycobacterium tuberculosis infection: implications for lifetime risk and population at-risk of tuberculosis disease. Proceedings of the Royal Society B **2021**; 288(1943):20201635.
28. Wong SH, Gao Q, Tsoi KKF, et al. Effect of immunosuppressive therapy on interferon γ release assay for latent tuberculosis screening in patients with autoimmune diseases: a systematic review and meta-analysis. Thorax **2016**; 71:64–72.
29. Yamasue M, Komiya K, Usagawa Y, et al. Factors associated with false negative interferon-γ release assay results in patients with tuberculosis: A systematic review with meta-analysis. Scientific Reports **2020**; 10:1607.
30. Baker H, Fine R, Suter F, et al. Implementation of a best practice advisory to improve infection screening prior to new prescriptions of biologics and targeted synthetic drugs. Arthritis Care & Research **2025**; 77:273–281.
31. Walsh AJ, Weltman M, Burger D, et al. Implementing guidelines on the prevention of opportunistic infections in inflammatory bowel disease. Journal of Crohn’s and Colitis **2013**; 7:e449–e456.
